# Recombinant human nerve growth factor (cenegermin) for moderate-to-severe dry eye: phase II, randomized, vehicle-controlled, dose-ranging trial

**DOI:** 10.1186/s12886-024-03564-w

**Published:** 2024-07-17

**Authors:** David Wirta, William Lipsky, Melissa Toyos, Joseph Martel, John Goosey, Anthony Verachtert, Sherif El-Harazi, Paul Karpecki, Marcello Allegretti, Giovanni Goisis, Georgea Pasedis, Flavio Mantelli

**Affiliations:** 1 Eye Research Foundation, 520 Superior Avenue, Suite 235, Newport Beach, CA 92663 USA; 2 Advanced Laser Vision & Surgical Institute and Intouch Clinical Research Center, 11550 Fuqua Street, Suite 250, Houston, TX 77034 USA; 3 Toyos Clinic, 2204 Crestmoor Road, Nashville, TN 37215 USA; 4 Martel Eye Medical Group, 11216 Trinity River Drive, Rancho Cordova, CA 95670 USA; 5 Houston Eye Associates, 2855 Gramercy Street, Houston, TX 77025 USA; 6 Moyes Eye Center, 5151 NW 88 Street, Kansas City, MO 64154 USA; 7 Global Research Management, 1510 S Central Avenue, Glendale, CA 91204 USA; 8 Kentucky Eye Institute, 601 Perimeter Drive, Suite 100, Lexington, KY 40517 USA; 9 Dompé farmaceutici S.p.A, Via Santa Lucia 6, Milan, 20122 Italy

**Keywords:** Cenegermin, Cornea, Dry eye disease, Nerve growth factor, SANDE, Schirmer test, Sjögren’s, Sjögren’s dry eye disease

## Abstract

**Background:**

Dry eye disease (DED) includes neurosensory abnormalities as part of its multifactorial etiology. Nerve growth factor is important for maintaining corneal nerve integrity and wound healing. Cenegermin (recombinant human nerve growth factor) is a topical biologic that promotes corneal healing in patients with neurotrophic keratitis. The purpose of this study was to evaluate efficacy and safety of cenegermin in moderate-to-severe DED and identify an optimal dosing strategy.

**Methods:**

This was a phase II, multicenter, randomized, double-masked, vehicle-controlled, dose-ranging clinical trial in patients with moderate-to-severe DED, including Sjögren’s DED (NCT03982368). Patients received 1 drop of cenegermin 3 times daily (t.i.d.; 20 mcg/mL), cenegermin 2 times daily (b.i.d.; 20 mcg/mL) and vehicle once daily, or vehicle t.i.d. for 4 weeks. Follow-up continued for 12 additional weeks. The primary endpoint was change in Schirmer I score from baseline to week 4. Other key endpoints included rate of responders (Schirmer I test > 10 mm/5 min) after treatment and change in Symptoms Assessment iN Dry Eye (SANDE) scores from baseline to end of follow-up. A 1-sided test (α = 0.025) was used to evaluate statistical significance.

**Results:**

At week 4, mean changes in Schirmer I scores were not statistically significantly different in either cenegermin group versus vehicle (cenegermin vs vehicle [treatment difference; 95% CI]: t.i.d., 2.60 mm and b.i.d., 3.99 mm vs 1.68 mm [t.i.d.: 0.93; −1.47 to 3.32, *P* = 0.078; b.i.d.: 2.31; −0.08 to 4.70, *P* = 0.066]). More patients responded to treatment with cenegermin t.i.d. and b.i.d. versus vehicle (t.i.d.: 25.9% [21/81, *P* = 0.028]; b.i.d.: 29.3% [24/82, *P* = 0.007] vs 11.9% [10/84]), with statistical significance (set at *P* < 0.025) observed in the b.i.d. group. Only cenegermin t.i.d. yielded statistically significant (*P* < 0.025) reductions in SANDE scores versus vehicle, which were sustained up to the end of follow-up (*P* value range, 0.002–0.008). Eye pain, primarily mild and transient, was the most frequently observed treatment-emergent adverse event with cenegermin. Similar results were observed in patients with Sjögren’s DED.

**Conclusions:**

Cenegermin was well tolerated and although this study did not meet its primary endpoint, significant improvement in patient-reported symptoms of dry eye was observed through follow-up. Larger studies evaluating cenegermin in patients with DED are warranted.

**Trial registration:**

NCT03982368; registered May 23, 2019.

**Supplementary Information:**

The online version contains supplementary material available at 10.1186/s12886-024-03564-w.

## Background

Dry eye disease (DED) is a multifactorial disease of the tears and ocular surface characterized by loss of tear film homeostasis and can be categorized on a continuum across aqueous-deficient and evaporative DED [1]. Moderate-to-severe DED can negatively impact daily activities, social and physical functioning, and quality of life, with worsening impact as disease severity increases [2–4]. If left untreated or in severe forms, DED can lead to vision-threatening complications including persistent epithelial defects, ulceration, corneal perforation, and, more rarely, loss of vision or even functional blindness [5].

Over the last decade, an emerging body of evidence has identified neurosensory abnormalities as part of the multifactorial etiology of DED, with other mechanisms including ocular surface inflammation and tear film instability [1]. Neurosensory abnormalities, such as atypical neuromodulation, disrupt tear secretion and subsequently ocular homeostasis [1, 6, 7]. Severe dry eye is often observed with Sjögren’s, and patients with Sjögren’s DED exhibit corneal epithelial, stromal, and neural abnormalities [5, 8–10]. Sjögren’s DED is associated with ocular discomfort and visual dysfunction, which can lead to corneal complications, such as corneal ulceration if left untreated [5, 11]. Furthermore, inflammation can have complex effects on neurotrophic factors present at the ocular surface [12]. Neurotrophic factors, such as nerve growth factor (NGF), are important for corneal nerve integrity and wound healing [12, 13]. Corneal nerves are responsible for corneal sensation, mediating blinking and tear reflexes in response to ocular surface damage [14–16], providing trophic support to ocular surface tissues, and helping maintain homeostasis of the ocular surface [15]. Thus, multiple mechanisms can disrupt homeostasis, resulting in adverse clinical and psychological outcomes for patients [5, 17].

Most therapies approved by the US Food and Drug Administration (FDA) for DED are designed to specifically target inflammatory mediators [18]. However, a therapy targeting the dynamic interplay between corneal epithelial cells and neurosensory abnormalities could be beneficial. Exogenous administration of recombinant human NGF (rhNGF) is of interest as a potential therapy for DED. NGF is an endogenous protein that is ubiquitous in the eye and known to modulate ocular inflammatory responses and promote tear secretion [14, 19]. Corneal epithelial cells and corneal nerve integrity rely on NGF: corneal nerves stimulate corneal epithelial and stromal cell proliferation, and these cells in turn release NGF to induce corneal nerve and stromal cell growth, support neuronal maturation and survival, and ultimately promote tissue maintenance and corneal wound healing [15, 20]. Improved corneal sensation and innervation have also been observed in NGF-treated patients with neurotrophic keratitis (also known as neurotrophic keratopathy) [21–24], a degenerative ocular disease characterized by impaired corneal sensitivity [25, 26]. While clinical data on the impact of cenegermin on corneal sensation and innervation in DED is limited, preclinical data suggests that topical NGF may increase corneal subbasal nerve densities as well as corneal sensitivity in the context of DED [27–29]. With its interplay between corneal epithelial, stromal cells, and corneal nerves, rhNGF could provide a multifaceted approach to treating DED.

Cenegermin (Oxervate, Dompé farmaceutici S.p.A., Italy; rhNGF) is a topical biologic administered as an eye drop and was approved by the FDA in 2018 for the treatment of neurotrophic keratitis. A phase IIa, open-label, multiple dose study established the use of cenegermin 20 mcg/mL in patients with moderate-to-severe DED [30]. Here we present findings from a phase II study evaluating the efficacy and safety of cenegermin eye drops administered for 4 weeks in patients with moderate-to-severe dry eye and the durability of treatment effect during 12 weeks of posttreatment follow-up. This dose-ranging study was designed to assess 2 dosing frequencies (b.i.d. and t.i.d.) to determine the optimal frequency based on safety and efficacy results. An exploratory analysis was also conducted to evaluate efficacy data in a subgroup of patients with Sjögren’s DED.

## Methods

### Study design

A phase II, multicenter, randomized, double-masked, vehicle-controlled, dose-ranging parallel group study was conducted at 11 sites in the United States (ClinicalTrials.gov, NCT03982368). The study was prospectively registered on May 23, 2019, and began on June 12, 2019. The study protocol was approved by the Advarra Institutional Review Board (registration number 00000971) at all study sites. Institutional Review Board approval was obtained for protocol amendments, informed consent forms, and any other relevant study-related documents at each study site. The study complied with the Declaration of Helsinki, relevant parts of the Code of Federal Regulations Title 21, and good clinical practice and good laboratory practice guidelines. Written informed consent was obtained from all patients before study initiation. This report adheres to CONSORT guidelines [31].

### Patients

Eligible patients were adults aged ≥ 18 years with moderate-to-severe DED diagnosed ≥ 6 months before enrollment, which was characterized by corneal and/or conjunctival staining with fluorescein using the National Eye Institute (NEI) grading system > 3; Symptoms Assessment iN Dry Eye (SANDE) questionnaire > 25 mm; Schirmer I test (without anesthesia) > 2 mm and < 10 mm/5 min; and tear film break-up time (TFBUT) < 10 s, all in the worse eye. Key inclusion criteria included best corrected distance visual acuity (BCDVA) score of ≥ 0.1 decimal units (20/200 Snellen value) in both eyes at study enrollment. For patients with Sjögren’s without an accompanying major rheumatic disease (per the American-European Consensus Group criteria for Sjögren’s), inclusion criteria were 4 of 6 total criteria or 3 of 4 clinical signs. Key exclusion criteria included evidence of an active ocular infection in either eye; presence of any other ocular disorder or condition requiring topical medication during the study; use of topical cyclosporine, corticosteroids, or other topical drug for the treatment of dry eye within 30 days before study enrollment; and ocular surgery within 90 days before the prescreening visit.

The Williams’ procedure was performed to evaluate the minimal effective daily dose of cenegermin eye drops [32, 33]. The sample size was determined by applying a published formula [34], with the probability level for a 1-sided test set at 0.025 and the power level at ~ 90%. Based on an analysis of a subset of hyposecretive patients from a clinical study of cenegermin in patients with dry eye [35], the difference in change from baseline between treatments was estimated as 5.3 mm with a standard deviation of 10.78. Owing to the COVID-19 pandemic, the sponsor stopped enrollment with 261 patients of the 300 originally planned, which was still adequate to observe the planned difference assumed for the minimum effective daily dose.

### Study procedures

One week after the screening visit, patients were randomized 1:1:1 to receive 1 drop of cenegermin 20 mcg/mL in both eyes t.i.d. (every 6 h), or 1 drop of cenegermin 20 mcg/mL b.i.d. (every 6 h) along with 1 drop of vehicle in both eyes once daily (q.d.) to match the t.i.d. dosing frequency, or 1 drop of vehicle t.i.d. in both eyes (every 6 h) for 4 weeks. Treatment consisted of cenegermin (Dompé farmaceutici S.p.A., Italy) and vehicle composed of a sterile solution for topical administration. The vials containing cenegermin or vehicle were identical in appearance, and the contents of the vials were indistinguishable. Each vial was marked to indicate the daily administration (eg, 1-morning, 2-afternoon, 3-evening). In addition, the vials were color-coded for each administration as follows: white = morning, yellow = afternoon, and light blue = evening. During the treatment period, 1 drop of topical lubricant preservative-free polyethylene glycol 400 0.25% was instilled in both eyes only if strictly needed by the patient; during the 12-week follow-up period, 1 drop of polyethylene glycol 400 0.25% was instilled in both eyes t.i.d., and additional drops were administered if strictly needed by the patient and documented in the patient’s diary. Details of the randomization process are located in Supplemental Materials.

### Endpoints and assessments

The primary endpoint was change from baseline in Schirmer I test at week 4. This test was performed at baseline and all study visits (weeks 2, 4, 8, 12, and 16). Rate of responders (defined as Schirmer I test value > 10 mm/5 min) at week 4 was also assessed via a preplanned sensitivity analysis.

Secondary endpoints included change from baseline to week 4 in the SANDE (global and frequency of dryness and/or irritation scores), the Impact of Dry Eye on Everyday Life (IDEEL), and the Patient Global Impression of Change (PGIC) questionnaire scores. Detailed descriptions of these patient-reported outcome measures are presented in Supplemental Materials. SANDE and IDEEL scores were assessed at baseline and all study visits. The PGIC questionnaire was conducted at weeks 4, 8, 12, and 16.

Additional secondary ocular endpoints included change from baseline to week 4 in corneal and conjunctival vital staining with fluorescein (NEI scales) and TFBUT, which were assessed at baseline and every study visit. Corneal and conjunctival vital staining total scores were the summation of corneal staining total score (calculated per graded scale of 0 to 3 applied central, superior, temporal, nasal, and inferior regions of the cornea for maximum score of 15) and conjunctival staining total score (calculated per graded scale of 0 to 3 applied to superior paralimbal, inferior paralimbal, and peripheral areas, and 0 to 9 applied to nasal and temporal conjunctiva, for a maximum score of 18). Other ocular endpoints included the Schirmer II test, which was assessed at baseline and study visits of weeks 4 and 16. A preplanned exploratory analysis was performed to evaluate the preliminary efficacy of cenegermin in patients with Sjögren’s DED.

Safety was analyzed as proportion of patients with treatment-emergent adverse events (TEAEs) assessed at every study visit. TEAEs were defined as any adverse event occurring or worsening on or after the first dose of study medication.

### Statistical analysis

Efficacy was analyzed in the full analysis set, which consisted of all randomized patients who received ≥ 1 dose of treatment and had ≥ 1 postbaseline efficacy measurement for the primary endpoint, and the per-protocol set, which consisted of all patients in the full analysis set who fulfilled the study protocol requirements in terms of treatment intake and collection of primary efficacy data with no major deviations; patients were analyzed according to the randomized treatment. Safety was assessed for all randomized patients who received ≥ 1 dose of treatment; patients were analyzed according to the treatment received.

Change from baseline was analyzed using analysis of variance, mixed model for repeated measures, or Fisher exact test when categorized; appropriate tests are described in the respective figure/table legends. The statistical threshold was set at 0.025 (1-sided) for all analyses, performed both in the overall population and the per-protocol population. To control for multiplicity, the analysis of the primary efficacy endpoint was performed sequentially according to Williams’ procedure [32, 33]. The first comparison compared cenegermin t.i.d. with vehicle. If the result was significant, the cenegermin b.i.d. dose was compared with vehicle; if it was not significant, then the test was stopped and descriptive comparisons of change from baseline for cenegermin t.i.d. and b.i.d. versus vehicle were evaluated. Additional details on statistical analysis are provided in Supplemental Materials.

## Results

### Demographics and baseline characteristics

**Fig. 1:**
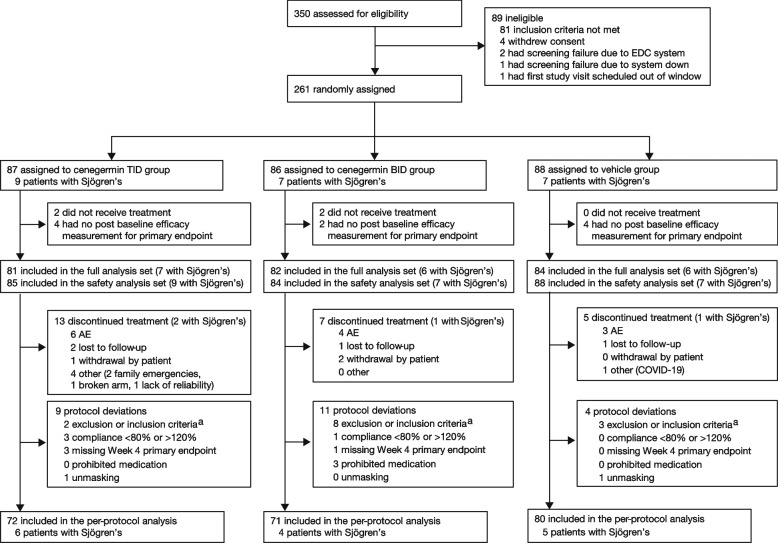
CONSORT diagram. AE, adverse event; b.i.d., 2 times daily; EDC, electronic data capture; SANDE, Symptoms Assessment iN Dry Eye; TFBUT, tear film break-up time; t.i.d., 3 times daily. ^a^Inclusion criteria not met: moderate-to-severe dry eye characterized by corneal and/or conjunctival staining with fluorescein using the National Eye Institute grading system > 3; SANDE questionnaire > 25 mm, Schirmer I test (without anesthesia) > 2 mm < 10 mm/5 min, and TFBUT < 10 s in the worse eye. Exclusion criteria met: use of topical cyclosporine, topical corticosteroids, or any other topical drug for treatment of dry eye in either eye within 30 days before study enrollment, or contact lenses or punctum plug use during the study

This study was conducted between June 12, 2019, and July 15, 2020; the last patient was enrolled on March 25, 2020. Of the 350 patients screened, a total of 261 patients met eligibility criteria and were randomized to receive cenegermin t.i.d. (*n* = 87), cenegermin b.i.d. (*n* = 86), or vehicle (*n* = 88; Fig. 1). Of the total randomized patients, 23 (8.8%) patients had Sjögren’s. Demographics and baseline characteristics were generally well balanced between groups (Table 1). Most enrolled patients were female (sex assigned at birth), with a median age of 61 years.

**Table 1:** Demographics and Baseline Characteristics

Characteristic	Cenegermin t.i.d. *N* = 81	Cenegermin b.i.d. *N* = 82	Vehicle *N* = 84
Age, median (range), years	62.0 (27–89)	61.0 (24–83)	61.0 (24–81)
Female, n (%)	57 (70.4)	69 (84.1)	72 (85.7)
Race, n (%)			
White	66 (81.5)	66 (80.5)	66 (78.6)
Asian	7 (8.6)	3 (3.7)	9 (10.7)
Black/African American	6 (7.4)	12 (14.6)	9 (10.7)
Other^a^	2 (2.5)	1 (1.2)	0
Eligible eye, n (%)			
Left	35 (43.2)	39 (47.6)	46 (54.8)
Right	46 (56.8)	43 (52.4)	38 (45.2)
Time since diagnosis of dry eyes, median (range), months	89.9 (7–530)	94.6 (12–511)	71.5 (9–419)
Baseline Schirmer I test, mean (SD), mm	5.3 (2.3)	4.9 (2.1)	4.7 (2.0)
Baseline global SANDE score, mean (SD)	73.9 (16.1)	73.4 (18.1)	76.3 (14.5)
Baseline severity of dryness and/or irritation SANDE scores, mean (SD)	71.5 (17.5)	71.4 (19.1)	74.1 (16.4)
Baseline IDEEL score, mean (SD)			
Daily activity limitations score	66.7 (20.5)	62.8 (20.1)	65.0 (22.6)
Observations, n	81	82	84
Emotional well-being score	68.0 (24.6)	64.2 (25.1)	66.3 (26.1)
Observations, n	81	82	84
Symptom bother score	63.6 (16.1)	65.1 (16.0)	62.9 (16.6)
Observations, n	81	82	84
Treatment satisfaction score	33.4 (20.9)	31.9 (21.3)	28.2 (20.8)
Observations, n	49	54	59
Treatment-related bother score	63.7 (21.6)	63.6 (23.8)	61.0 (24.3)
Observations, n	55	63	61
Work limitations score	66.1 (28.7)	58.5 (24.0)	56.5 (28.6)
Observations, n	46	43	54
Baseline TFBUT score, mean (SD)	4.4 (1.5)	4.3 (1.8)	4.6 (2.1)
Baseline corneal and conjunctiva vital staining with fluorescein score, mean (SD)	14.9 (5.3)	14.0 (6.7)	15.5 (5.7)

*b.i.d.* 2 times daily, *IDEEL* Impact of Dry Eye on Everyday Life, *SANDE* Symptom Assessment iN Dry Eye, *SD* standard deviation, *TFBUT* tear film break-up time, *t.i.d.* 3 times daily

^a^Other includes American Indian or Alaskan native, Native Hawaiian, or other Pacific Islander

### Primary efficacy analyses

**Fig. 2:**
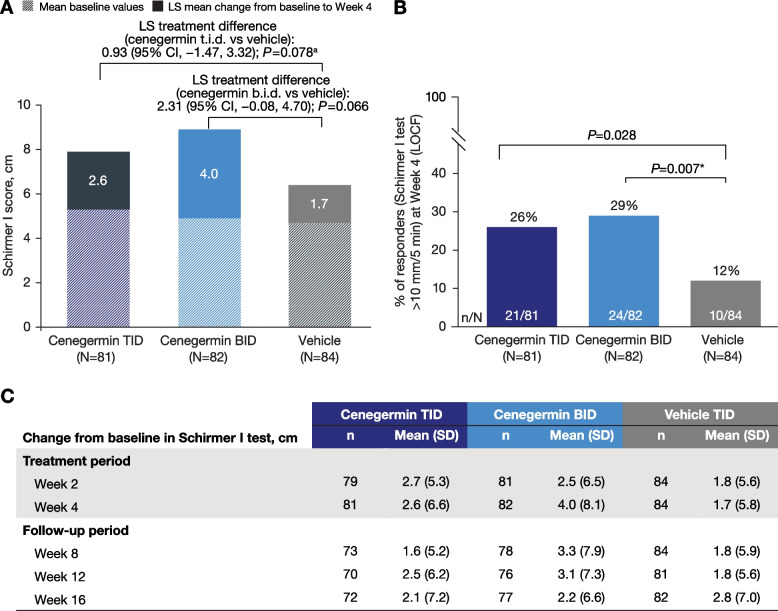
Summary of Schirmer I test outcomes. **A** LS mean change in Schirmer I test from baseline to week 4, **B** mean change from baseline across all study visits, and (**C**) analyzed as rate of responders at week 4 (full analysis set). b.i.d., 2 times daily; LOCF, last observation carried forward; LS, least squares; t.i.d., 3 times daily. The changes from baseline in Schirmer I test were analyzed using analysis of variance (including treatment as the main factor), followed by preplanned comparison from vehicle and cenegermin dosages according to Williams’ procedure. A Fisher exact test was applied in the analysis of patients categorized as responders on the Schirmer I test. *Denotes *P* < 0.025. ^a^If cenegermin t.i.d. was different from vehicle, then the cenegermin b.i.d. dose was compared with vehicle; because significance was not observed, the *P* value for cenegermin b.i.d. versus vehicle is presented only for descriptive purposes

In the primary endpoint analysis with the full analysis set, least squares (LS) mean change from baseline to week 4 in Schirmer I test was not significantly different in the cenegermin groups compared with vehicle (cenegermin t.i.d. vs vehicle [treatment difference; 95% CI]: 2.60 vs 1.68 mm [0.93; − 1.47 to 3.32], *P* = 0.078; cenegermin b.i.d. vs vehicle: 3.99 vs 1.68 mm [2.31; − 0.08 to 4.70], *P* = 0.066; Fig. 2A). An adjusted analysis confirmed these results (cenegermin t.i.d. vs vehicle [treatment difference; 95% CI]: 2.22 vs 1.40 mm [0.82; − 1.60 to 3.24], *P* = 0.088; cenegermin b.i.d. vs vehicle: 3.68 vs 1.40 mm [2.29; − 0.11 to 4.68], *P* = 0.074; Supplemental Figure S1). Analyses for the primary endpoint using the per-protocol set were consistent with findings in the full analysis set (cenegermin t.i.d. vs vehicle: 2.99 vs 1.28 mm [1.71; − 0.70 to 4.12], *P* = 0.032; cenegermin b.i.d. vs vehicle: 3.72 vs 1.28 [2.44; 0.02 to 4.86], *P* = 0.029). During the follow-up period, mean change from baseline in Schirmer I test scores was generally higher with cenegermin treatment compared with vehicle through week 12 although not statistically significantly different; results were similar across groups by week 16 (Fig. 2B).

At week 4, the rate of responders on the Schirmer I test (> 10 mm/5 min) in the full analysis set was significantly higher in the cenegermin b.i.d. versus vehicle group (29.3% [24/82] vs 11.9% [10/84], *P* = 0.007); the rate of responders in the cenegermin t.i.d. group was also higher when compared with vehicle (25.9% [21/81] versus 11.9% [10/84], *P* = 0.028; Fig. 2C), and not significantly different than cenegermin b.i.d. In the per-protocol set, the rate of responders was significantly higher with the *P* value set < 0.025 in both cenegermin groups compared with vehicle (cenegermin t.i.d. vs vehicle: 29.2% [21/72] vs 8.8% [7/80], *P* = 0.001; cenegermin b.i.d. vs vehicle: 28.2% [20/71] vs 8.8% [7/80], *P* = 0.003).

### Secondary efficacy analyses

**Fig. 3:**
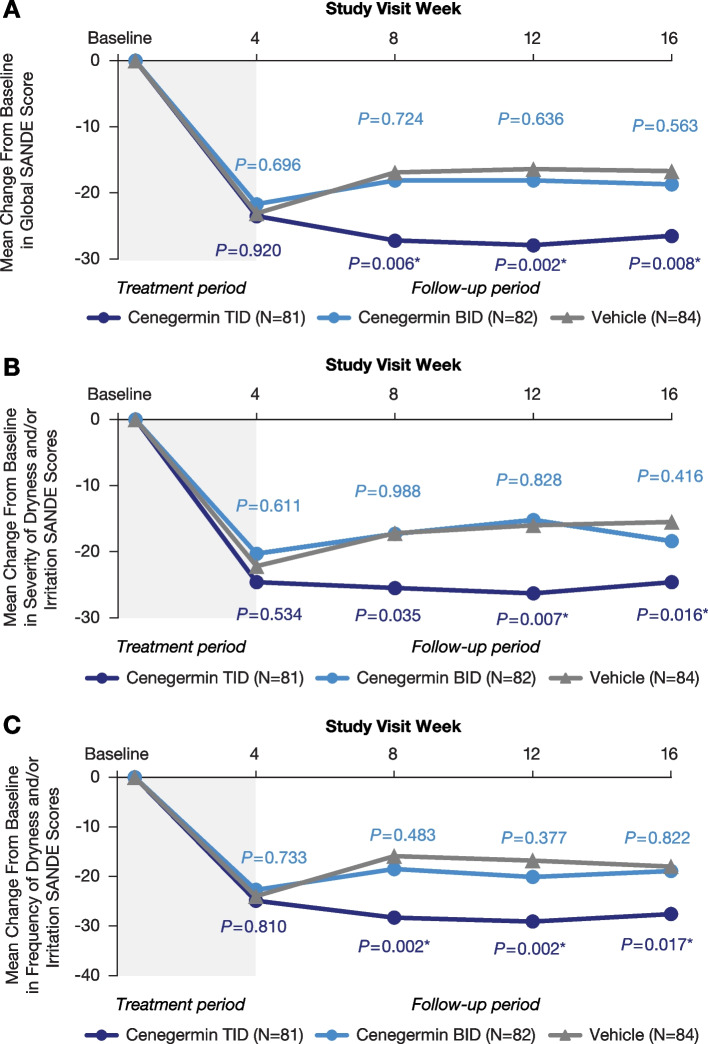
Summary of SANDE questionnaire outcomes. Change from baseline in (**A**) global, (**B**) severity of dryness and/or irritation, and (**C**) frequency of dryness and/or irritation SANDE scores (full analysis set). b.i.d., 2 times daily; SANDE, Symptoms Assessment iN Dry Eye; t.i.d., 3 times daily. SANDE scores for each question range from 0 mm (frequency is “rarely” and severity is “very mild”) to 100 mm (frequency is “all of the time” and severity is “very severe”), and the global SANDE score is calculated as the frequency score multiplied by the severity score and obtaining the square root. The changes from baseline in SANDE scores were analyzed at each time point using a *t* test for the comparison of cenegermin t.i.d. and b.i.d. versus vehicle. *Denotes *P* < 0.025

**Fig. 4:**
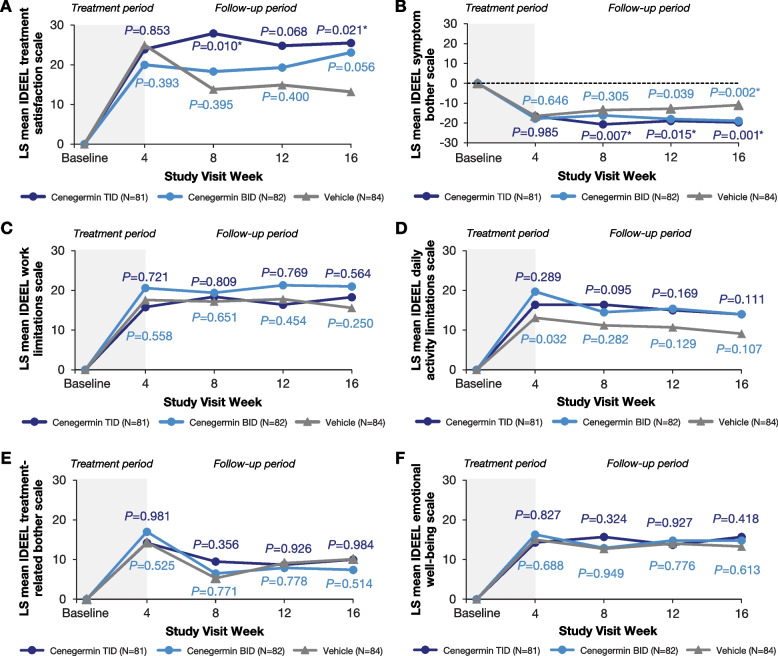
Summary of IDEEL questionnaire outcomes. Change from baseline in IDEEL questionnaire on (**A**) treatment satisfaction, (**B**) symptom bother, (**C**) work limitations, (**D**) daily activity limitations, (**E**) treatment-related bother, and (**F**) emotional well-being (full analysis set). b.i.d., 2 times daily; IDEEL, Impact of Dry Eye on Everyday Life; LS, least squares; t.i.d., 3 times daily. Change from baseline in each IDEEL scale score (1 score for each questionnaire module) was analyzed using a mixed model for repeated measures, with fixed, categorical effects of treatment (cenegermin t.i.d., cenegermin b.i.d., and vehicle t.i.d.), visit (weeks 4, 8, 12, and 16), and treatment by visit interaction. A 4- or 5-point Likert scale was used for each IDEEL question, with the exception of yes/no questions. Patient was considered as a random effect, and the covariance matrix used was “unstructured.” *Denotes *P* < 0.025

In global, severity, and frequency of dryness and/or irritation SANDE scores, mean change from baseline to week 4 was not significantly different between the cenegermin groups and the vehicle group (Fig. 3A-C). However, during the follow-up period, the cenegermin t.i.d. group had significantly greater improvement in symptoms compared with vehicle that were generally stable for up to 16 weeks after treatment initiation. Similarly, significant improvements were observed during the follow-up period in the cenegermin t.i.d. group versus the vehicle group in treatment satisfaction at weeks 8 and 16 (Fig. 4A) and symptoms at weeks 8, 12, and 16 (Fig. 4B) based on the IDEEL questionnaire; other quality-of-life IDEEL scores were comparable between cenegermin b.i.d., t.i.d., and vehicle groups throughout the follow-up period (Fig. 4C-F). In the PGIC questionnaire, proportion of patients with improved scores (minimally improved, much improved, or very much improved) was significantly higher in the cenegermin t.i.d. group, but not in the cenegermin b.i.d. group, compared with vehicle at weeks 8 and 16 (Supplemental Table S1).

Least squares mean change from baseline to week 4 in Schirmer II test was significantly greater with cenegermin t.i.d., but not cenegermin b.i.d., compared with vehicle (cenegermin t.i.d. vs vehicle [treatment difference; 95% CI]: 2.19 vs 0.21 mm [1.97; − 0.19 to 4.13], *P* = 0.025; cenegermin b.i.d. vs vehicle: 0.89 vs 0.21 mm [0.68; − 1.48 to 2.83], *P* = 0.243). Mean change from baseline in TFBUT was significantly greater with cenegermin t.i.d., but not cenegermin b.i.d., compared with vehicle at week 4, but not during the follow-up period (Supplemental Figure S2). No significant differences were observed with either treatment group compared with vehicle in mean change from baseline to any study visit in combined corneal and conjunctival vital staining scores (Supplemental Figure S3).

### Safety

Eye pain was the most common ocular TEAE during the treatment period and was mild and transient, with only 1 patient per group reporting eye pain during the follow-up period (Table 2). Rates of discontinuation because of eye pain were low and similar across the cenegermin and vehicle groups. Moderate TEAEs of eye pain were observed in 3 patients in each of the cenegermin t.i.d. and b.i.d. groups (none were observed in the vehicle group); severe TEAEs of eye pain were observed in 1 patient in each of the cenegermin t.i.d. and vehicle groups. No moderate or severe TEAEs of eye pain were observed during the follow-up period. There were no discernable differences in ocular TEAEs reported in the cenegermin t.i.d. and b.i.d. groups. Fewer ocular TEAEs were reported in each treatment group in the follow-up period compared with the treatment period, and there was no discernable pattern across treatment groups. No treatment-emergent serious adverse events (SAEs) potentially related to the study drug were reported during the treatment or follow-up periods.

**Table 2:** Summary of TEAEs During the Treatment and Follow-up Periods

	Treatment period			Follow-up period		
n (%)	Cenegermin t.i.d. (*N* = 85)	Cenegermin b.i.d. (*N* = 84)	Vehicle (*N* = 88)	Cenegermin t.i.d. (*N* = 85)	Cenegermin b.i.d (*N* = 84)	Vehicle (*N* = 88)
Any TEAE	65 (76.5)	60 (71.4)	26 (29.5)	19 (22.4)	19 (22.6)	18 (20.5)
Any ocular TEAE	61 (71.8)	60 (71.4)	20 (22.7)	9 (10.6)	14 (16.7)	11 (12.5)
Ocular TEAEs occurring in ≥ 5% of patients in any group						
Dry eye	1 (1.2)	3 (3.6)	5 (5.7)	2 (2.4)	0	1 (1.1)
Eye irritation	2 (2.4)	5 (6.0)	5 (5.7)	1 (1.2)	2 (2.4)	4 (4.5)
Eye pain	54 (63.5)	42 (50.0)	4 (4.5)	1 (1.2)	1 (1.2)	1 (1.1)
Eyelid pain	3 (3.5)	9 (10.7)	0	0	0	0
Ocular discomfort	6 (7.1)	4 (4.8)	0	1 (1.2)	0	0
Photophobia	6 (7.1)	4 (4.8)	0	0	2 (2.4)	0
Swelling of eyelid	1 (1.2)	6 (7.1)	1 (1.1)	1 (1.2)	0	0
Any TEAE leading to discontinuation	5 (5.9)	2 (2.4)	2 (2.3)	1 (1.2)	1 (1.2)	1 (1.1)
TEAE leading to discontinuation in > 1 patient in any group						
Eye pain	4 (4.7)	2 (2.4)	2 (2.3)	0	0	0
Headache	2 (2.4)	0	0	0	0	0
Any potentially related TEAE	60 (70.6)	55 (65.5)	13 (14.8)	2 (2.4)	2 (2.4)	5 (5.7)
Any serious potentially related TEAE	0	0	0	0	0	0

*b.i.d.* 2 times daily, *TEAE* treatment-emergent adverse event, *t.i.d.* 3 times daily

Of note, the proportion and frequency of preservative-free artificial tears used during the study was comparable across all treatment groups, with a greater proportion of patients using ≥ 5 drops per day during the follow-up period compared with the treatment period (Supplemental Table S2).

### Subgroup analysis in patients with Sjögren’s

Primary and key secondary efficacy outcomes among patients with Sjögren’s DED were generally consistent with patients with non-Sjögren’s DED (Supplemental Table S3). When compared with vehicle at week 16, both global and severity of dryness and/or irritation SANDE scores for the cenegermin t.i.d. group significantly improved in patients with Sjögren’s DED (global, *P* = 0.011; severity, *P* = 0.002) and improved trending toward significance in patients with non-Sjögren’s DED (global, *P* = 0.038; severity, *P* = 0.081). At week 16, IDEEL symptom bother improved in both cenegermin t.i.d. subgroups compared with vehicle, with significant improvements in patients with non-Sjögren’s DED (*P* = 0.006) and improvements trending toward significant in patients with Sjögren’s DED (*P* = 0.027; Supplemental Table S4). Similarly, safety profiles among patients with Sjögren’s DED were generally consistent with patients with non-Sjögren’s DED, with few instances of TEAEs leading to discontinuation and no serious TEAEs in either subgroup (Supplemental Table S5).

## Discussion

After 4 weeks of treatment, no statistically significant differences in tear production, assessed by Schirmer I test, were observed in patients with moderate-to-severe dry eye treated with cenegermin compared with vehicle. Thus, the study did not meet the primary endpoint. However, the rates of responders on Schirmer I test (defined as Schirmer I > 10 mm/5 min) at the end of the treatment period were higher in the cenegermin groups compared with vehicle. Significant improvements in patient-reported symptoms of dry eye specifically with the t.i.d. dosing strategy were observed and sustained for up to 16 weeks after treatment initiation. Overall, cenegermin was well tolerated. Eye pain was the most frequently observed TEAE, which was primarily mild and transient. Study discontinuation was low and there were no SAEs related to study drug during treatment or follow-up.

In comparison with vehicle-treated patients, patients treated with cenegermin had transient improvements in tear film stability assessed by TFBUT. While cenegermin favorably impacted corneal and conjunctival staining, specifically in the t.i.d. group, the changes observed were not statistically significant. The differential effects of cenegermin on corneal staining in patients with neurotrophic keratitis compared with patients with DED may be explained by the different underlying pathophysiologies responsible for corneal staining in each of these diseases. The mechanisms inducing corneal staining vary considerably between neurotrophic keratitis and DED. If corneal staining is due to ocular inflammation, such as that seen in DED, then cenegermin may not lead to a pronounced improvement in staining, particularly if the underlying inflammation is not addressed and resolved [36]. On the other hand, corneal staining in neurotrophic keratitis is, in part, attributable to lack of corneal nerve–derived trophic support to the corneal epithelium [37]. Accordingly, cenegermin, a recombinant human nerve growth factor, has been shown to have a notable impact on ocular surface staining in patients with neurotrophic keratitis [25].

Improvements in patient-reported wellness questionnaires increased during the follow-up period and were sustained for up to 12 weeks after the completion of cenegermin treatment, specifically with the t.i.d. dosing regimen. Notably, patients first demonstrated an improvement in clinical signs (Schirmer I test, week 4) followed by a gradual but consistent and statistically significant improvement in symptoms in the t.i.d. group during the follow-up period, which was seen as early as 4 weeks after completion of the treatment (week 8). This is consistent with what is usually observed in the treatment of DED, where symptomatic improvement lags behind improvement in clinical signs [38]. While increased tear secretion may be associated with drug intolerance in certain contexts, our data do not suggest that the observed increases in Schirmer I scores in this study were due to intolerance to cenegermin because (a) there was accompanying improvement in clinical symptoms and (b) tear production remained increased compared with baseline even after treatment was completed and cenegermin was no longer being used (eg, during the follow-up period up to week 12 in the cenegermin b.i.d. group). DED is known to have a discordance between clinical signs and the frequency of symptoms, which can also explain the patient-reported symptom improvements observed throughout the follow-up period [39]. Nerve growth factor has been shown to increase tear production, goblet cell density, mucin secretion, and epithelial proliferation, all of which may contribute to symptom improvement in DED [29, 40, 41]. The pattern of symptom improvement in the cenegermin t.i.d. group in this study may be explained by a mechanism of action in line with that of cenegermin in a symptomatic disease such as DED. In this and previous studies, eye pain has been the most frequently observed treatment-emergent adverse event associated with cenegermin therapy, presumably because of its effects on nociceptor sensitization, and is almost exclusively reported during the treatment period. Therefore, although symptoms improved for patients in all 3 groups between baseline and the end of the 4-week treatment period, reported symptom improvement in the cenegermin b.i.d. and t.i.d. groups may have been counteracted by the onset of eye pain in some patients. During the 12-week follow-up period, when eye pain was rarely reported, symptom improvement in the vehicle and cenegermin b.i.d. groups regressed slightly while the cenegermin t.i.d. group demonstrated a continued and sustained improvement. This suggests that the higher concentration of cenegermin provided by the t.i.d. dosing regimen may have induced greater homeostasis of the ocular surface resulting in greater improvement and durability of improvement in symptoms. Moreover, because DED has multifactorial etiologies involving neurosensory abnormalities, tear film instability, and inflammation [1], heterogeneity could exist in the patient population of the present study and could partly explain the variable outcomes in signs and symptoms. Therefore, investigation into the efficacy and safety of cenegermin in patients with varying dry eye severity, including mild DED, may be warranted.

A small subgroup of patients in this study had Sjögren’s DED. In our study, efficacy results from an exploratory analysis in patients with Sjögren’s DED were comparable to those in patients with non-Sjögren’s DED. However, the low number of patients in the subgroup with Sjögren’s DED prevents drawing conclusions about the effects of cenegermin in this patient population. The recently completed phase III PROTEGO-1 and PROTEGO-2 randomized clinical trials evaluated efficacy and safety of cenegermin specifically in patients with severe Sjögren’s DED (ClinicalTrials.gov identifiers, NCT05136170 and NCT05133180) using the t.i.d. dosing regimen established in this study.

Consistent with previous cenegermin studies [25, 30], eye pain was the most frequently observed TEAE in this study, which may be explained by the complex role of NGF in neural pathways related to nociception [42]. In general, eye pain was mild and transient, and did not continue into the follow-up period. Moreover, eye pain did not appear to affect patient satisfaction with treatment, their reported increased quality of life, or their ability to complete the 4-week treatment. Indeed, despite the incidence of eye pain during the treatment period only, patients continued to report improvements across the SANDE, IDEEL, and PGIC questionnaires throughout the follow-up period. These results suggest that cenegermin had a lasting effect beyond the treatment period, supporting the results from a study in patients with moderate-to-severe neurotrophic keratitis showing corneal reinnervation and nerve growth increase past the treatment period [24].

This study has some limitations. While the demographics of the trial population were unbalanced, they reflected the general population with DED (ie, predominantly older and women). Although sex at birth was reported, no analyses comparing the effect of cenegermin between sexes were carried out. Additionally, results observed in the per-protocol analysis set were slightly better than those in the full analysis set, demonstrating that adherence to the protocol may affect efficacy, which might also affect expected results from real-life usage. However, these limitations, as well as a possible heterogeneity, will be addressed by the 2 ongoing phase III trials powered to detect treatment effects specifically in patients with severe Sjögren’s DED. Of note in this phase II trial, patients with Sjögren’s DED randomized to the vehicle group showed no improvement in Schirmer I and II tests.

## Conclusions

Although cenegermin did not meet the primary efficacy endpoint, it transiently improved aqueous tear production and showed significant improvement in patient-reported symptoms of dry eye after treatment completion, which were sustained for 16 weeks after the initiation of treatment. This dose-ranging study also effectively identified cenegermin t.i.d. as the optimal dosing regimen based on safety and efficacy results, as it provided a significant and durable improvement in dry eye symptoms without an increase in adverse events compared with cenegermin b.i.d. These results build upon previous studies evaluating efficacy and safety of cenegermin for neurotrophic keratitis and moderate-to-severe dry eye. Future studies will expand on these findings by evaluating cenegermin t.i.d. in a larger population of patients with severe Sjögren’s DED.

### Supplementary Information

Supplementary Material 1.

Supplementary Material 2.

Supplementary Material 3.

Supplementary Material 4.

Supplementary Material 5.

## Data Availability

Dompé is committed to responsible data sharing regarding the clinical trials we sponsor. This includes clinical trial registration and results information submission requirements described in Sect. 801 of the Food and Drug Administration Amendments Act of 2007, known as FDAAA 801, which will be available on ClinicalTrials.gov. This clinical trial data can be requested by any qualified researchers or physicians and Dompé will evaluate the data request on a case-by-case basis. Data requests can be submitted at any time to usmedinfo@dompe.com.

## Abbreviations

BCDVA: Best corrected distance visual acuity
b.i.d.: 2 Times daily
DED: Dry eye disease
FDA: US Food and Drug Administration
IDEEL: Impact of Dry Eye on Everyday Life
LS: Least squares
NEI: National Eye Institute
NGF: Nerve growth factor
PGIC: Patient Global Impression of Change
q.d.: Once daily
SAE: Severe adverse event
SANDE: Symptoms Assessment iN Dry Eye
TEAE: Treatment-emergent adverse event
TFBUT: Tear film break-up time
t.i.d.: 3 Times daily
