# Supplementary material for: Recombinant human nerve growth factor (cenegermin) for moderate-to-severe dry eye: phase II, randomized, vehicle-controlled, dose-ranging trial
Source: BMC Ophthalmol. 2024 Jul 17;24:290. doi: 10.1186/s12886-024-03564-w (PMC11253442; doi:10.1186/s12886-024-03564-w)
Supplement: Supplementary file 1 — Supplementary Material 1. [file 12886_2024_3564_MOESM1_ESM.docx]

**SUPPLEMENTAL MATERIALS**

**Randomization process**

Each randomized patient was allocated a randomization number, according to the stratified randomization list; using an interactive web response system, randomization was stratified by presence or absence of Sjögren’s, and dropouts after randomization were not replaced. Patients were assigned to treatment in numerical order. A tear-off label from the kit box, with the kit number, was attached to the investigational medicinal product dispensing log. The randomization list and the list of kit numbers were generated by a member of the Syneos Health Biostatistics department who was not involved in the conduct of the study. The vials containing rhNGF or vehicle were identical in appearance, and the contents of the vials were indistinguishable. All patients, investigators, site staff, and sponsor’s clinical research personnel remained masked until the unmasking at the final statistical analysis after database lock, except in case of specific events that required unmasking of the patient; unmasking events were recorded and reported.

**Quality-of-life tests**

The Symptoms Assessment iN Dry Eye (SANDE) questionnaire comprises 2 questions evaluating the frequency and severity of eyes feeling dry, irritated, or both, with scores for each question ranging from 0 mm (frequency is “rarely” and severity is “very mild”) to 100 mm (frequency is “all of the time” and severity is “very severe”). The global SANDE score is calculated as the frequency score multiplied by the severity score and obtaining the square root. The Impact of Dry Eye on Everyday Life (IDEEL) questionnaire comprises 3 modules and 57 questions evaluating the effects of dry eye disease on quality of life, treatment satisfaction over the past 2 weeks, and dry eye symptoms, with a higher score indicative of greater impact on the measured outcome (lower bother score is reflective of less bothersome symptoms). A 4- or 5- point Likert scale was used for each question, with the exception of yes/no questions (1). In the Patient Global Impression of Change questionnaire (PGIC), using a 7-point scale (1 = very much improved; 7 = very much worse), patients report perceived degree of change in the general state of their health relative to the start of the study.

**Statistics**

Schirmer I test was analyzed using an analysis of variance with treatment as the main factor with preplanned comparisons of cenegermin t.i.d. and b.i.d. versus vehicle according to Williams’ procedure (2, 3). Comparisons were performed sequentially to avoid issues with multiplicity. The first comparison evaluated the cenegermin t.i.d. dose with vehicle. In case of a significant result, the cenegermin b.i.d. dose was compared with vehicle; if the result was not significant, then the test was stopped and descriptive comparisons of change from baseline for cenegermin t.i.d. and b.i.d. versus vehicle were evaluated using a *t* test at each time point. An explorative sensitivity analysis of the primary endpoint was also conducted, including in the model the absence/presence of diagnosis of Primary Sjogren’s Syndrome and its interaction with treatments as covariates. The proportion of responders at week 4, defined as a Schirmer I test >10 mm/5 minutes, was also evaluated using a non-parametric Fisher exact test in a sensitivity analysis.

Change from baseline to week 4 in SANDE, Schirmer II test, TFBUT, and corneal and conjunctival vital staining score was analyzed in a similar manner as for the primary endpoint. For each of these endpoints, descriptive comparisons were also provided using a *t* test at each time point.

For Schirmer I test, Schirmer II test, SANDE, fluorescein staining, and TFBUT, the last observation carried forward method was used for imputing missing data in the full analysis set at week 4.

Change from baseline in each IDEEL scale score (1 score for each questionnaire module) was analyzed using a mixed model for repeated measures, with fixed, categorical effects of treatment (cenegermin t.i.d., cenegermin b.i.d., and vehicle t.i.d.), visit (weeks 4, 8, 12, and 16), and treatment by visit interaction. Patient was considered as a random effect, and the covariance matrix used was “unstructured.”

Because of the ordinal nature of the PGIC questionnaire, *P* values were assessed using Wilcoxon rank sum test comparing vehicle with cenegermin t.i.d. and b.i.d. For the PGIC questionnaire, summary of responses by treatment and visit were recorded.

In general, continuous variables were summarized using number of observations, number of missing data (when applicable), mean, standard deviation, median, minimum, and maximum. Categorical variables were summarized using number of observations, number of missing data (when applicable), and frequency and percentages of patients. The 95% and 99% confidence limits for the mean were calculated based on the t-distribution, where indicated. The denominator was based on the total number of patients in the treatment group or overall, or on the total number of patients with non-missing data, as appropriate.

**References**

1. Abetz L, Rajagopalan K, Mertzanis P, Begley C, Barnes R, Chalmers R. Development and validation of the impact of dry eye on everyday life (IDEEL) questionnaire, a patient-reported outcomes (PRO) measure for the assessment of the burden of dry eye on patients. Health Qual Life Outcomes. 2011;9:111.

2. Williams DA. The comparison of several dose levels with a zero dose control. Biometrics. 1972;28:519-31.

3. Williams DA. A test for differences between treatment means when several dose levels are compared with a zero dose control. Biometrics. 1971;27:103-17.
