# Supplementary material for: Recombinant human nerve growth factor (cenegermin) for moderate-to-severe dry eye: phase II, randomized, vehicle-controlled, dose-ranging trial
Source: BMC Ophthalmol. 2024 Jul 17;24:290. doi: 10.1186/s12886-024-03564-w (PMC11253442; doi:10.1186/s12886-024-03564-w)
Supplement: Supplementary file 2 — Supplementary Material 2. [file 12886_2024_3564_MOESM2_ESM.docx]

**SUPPLEMENTAL TABLES**

| **Supplemental Table S1.** Proportion of Patients Reporting Improvements in PGIC by Study Visit (Full Analysis Set)^a^ | | | |
| --- | --- | --- | --- |
| **n/N (%)** | **Cenegermin  t.i.d.**  **(N=81)** | **Cenegermin b.i.d.**  **(N=82)** | **Vehicle**  **(N=84)** |
| Week 4 |  |  |  |
| Very much improved | 11/78 (14.1) | 2/81 (2.5) | 4/84 (4.8) |
| Much improved | 18/78 (23.1) | 15/81 (18.5) | 20/84 (23.8) |
| Minimally improved | 18/78 (23.1) | 21/81 (25.9) | 20/84 (23.8) |
| No change | 24/78 (30.8) | 38/81 (46.9) | 38/84 (45.2) |
| Minimally worse | 6/78 (7.7) | 4/81 (4.9) | 2/84 (2.4) |
| Much worse | 1/78 (1.3) | 1/81 (1.2) | 0 |
| Very much worse | 0 | 0 | 0 |
| *P* value^a^ | 0.302 | 0.244 |  |
| Week 8 |  |  |  |
| Very much improved | 9/74 (12.2) | 3/78 (3.8) | 1/84 (1.2) |
| Much improved | 23/74 (31.1) | 11/78 (14.1) | 16/84 (19.0) |
| Minimally improved | 19/74 (25.7) | 21/78 (26.9) | 17/84 (20.2) |
| No change | 21/74 (28.4) | 37/78 (47.4) | 46/84 (54.8) |
| Minimally worse | 1/74 (1.4) | 4/78 (5.1) | 2/84 (2.4) |
| Much worse | 0 | 1/78 (1.3) | 2/84 (2.4) |
| Very much worse | 1/74 (1.4) | 1/78 (1.3) | 0 |
| *P* value^a^ | 0.000 | 0.885 |  |
| Week 12 |  |  |  |
| Very much improved | 11/73 (15.1) | 3/77 (3.9) | 4/82 (4.9) |
| Much improved | 16/73 (21.9) | 10/77 (13.0) | 12/82 (14.6) |
| Minimally improved | 22/73 (30.1) | 24/77 (31.2) | 24/82 (29.3) |
| No change | 15/73 (20.5) | 36/77 (46.8) | 40/82 (48.8) |
| Minimally worse | 5/73 (6.8) | 1/77 (1.3) | 0 |
| Much worse | 2/73 (2.7) | 2/77 (2.6) | 2/82 (2.4) |
| Very much worse | 2/73 (2.7) | 1/77 (1.3) | 0 |
| *P* value^a^ | 0.035 | 0.698 |  |
| Week 16 |  |  |  |
| Very much improved | 7/75 (9.3) | 3/78 (3.8) | 6/82 (7.3) |
| Much improved | 24/75 (32.0) | 19/78 (24.4) | 16/82 (19.5) |
| Minimally improved | 22/75 (29.3) | 20/78 (25.6) | 19/82 (23.2) |
| No change | 19/75 (25.3) | 33/78 (42.3) | 36/82 (43.9) |
| Minimally worse | 2/75 (2.7) | 2/78 (2.6) | 5/82 (6.1) |
| Much worse | 1/75 (1.3) | 0 | 0 |
| Very much worse | 0 | 1/78 (1.3) | 0 |
| *P* value^a^ | 0.016 | 0.706 |  |
| b.i.d., 2 times daily; PGIC, Patient Global Impression of Change; t.i.d., 3 times daily. ^a^*P* value based on Wilcoxon rank sum test; significance threshold was *P*<0.025. | | | |

| **Supplemental Table S2.** Proportion and Frequency of Preservative-Free Artificial Tears Use by Period (FAS) | | | |
| --- | --- | --- | --- |
| **n (%)** | **Cenegermin  t.i.d.**  **N=81** | **Cenegermin b.i.d.**  **N=82** | **Vehicle**  **N=84** |
| **During treatment period^a^** | | | |
| 0 to <1 | 41 (50.6) | 41 (50.0) | 50 (59.5) |
| ≥1 drop/day but <2 | 4 (4.9) | 9 (11.0) | 9 (10.7) |
| ≥2 drop/day but <3 | 8 (9.9) | 5 (6.1) | 5 (6.0) |
| ≥3 drop/day but <4 | 8 (9.9) | 4 (4.9) | 3 (3.6) |
| ≥4 drop/day but <5 | 5 (6.2) | 8 (9.8) | 9 (10.7) |
| ≥5 drop/day but <6 | 1 (1.2) | 3 (3.7) | 2 (2.4) |
| ≥6 drop/day | 14 (17.3) | 12 (14.6) | 6 (7.1) |
| **During follow-up period^b^** | | | |
| 0 to <1 | 5 (6.2) | 4 (4.9) | 6 (7.1) |
| ≥1 drop/day but <2 | 0 | 1 (1.2) | 1 (1.2) |
| ≥2 drop/day but <3 | 2 (2.5) | 2 (2.4) | 0 |
| ≥3 drop/day but <4 | 0 | 1 (1.2) | 1 (1.2) |
| ≥4 drop/day but <5 | 1 (1.2) | 3 (3.7) | 0 |
| ≥5 drop/day but <6 | 21 (25.9) | 24 (29.3) | 23 (27.4) |
| ≥6 drop/day | 52 (64.2) | 47 (57.3) | 53 (63.1) |
| b.i.d., twice daily; FAS, full analysis set; t.i.d., 3 times daily. ^a^Drop/day during the treatment period = sum of drops during treatment period/(date of week 4 or date of last visit during treatment period − date of baseline + 1). ^b^Drop/day during the follow-up period = sum of drops during follow-up period/(date of week 16 or date of last visit during follow-up period − date of week 4 + 1). | | | |

| **Supplemental Table S3.** Primary and Select Secondary Efficacy Outcomes by Presence of Sjögren’s | | | | | | |
| --- | --- | --- | --- | --- | --- | --- |
|  | **Patients with Sjögren’s dry eye disease** | | | **Patients with non-Sjögren’s dry eye disease** | | |
|  | **Cenegermin t.i.d.**  **N=7** | **Cenegermin b.i.d.**  **N=6^a^** | **Vehicle**  **N=6** | **Cenegermin t.i.d.**  **N=74** | **Cenegermin b.i.d.**  **N=76** | **Vehicle**  **N=78** |
| **Primary, mean (SD) Schirmer I test score, mm** | | | | | | |
| Baseline | 4.6 (2.4) | 3.0 (1.9) | 4.0 (1.3) | 5.4 (2.2) | 5.0 (2.1) | 4.7 (2.0) |
| Change from baseline to week 4 (LOCF) | 2.0 (3.9)  *P=*0.339 | 3.5 (5.3)  *P=*0.196 | 0.0 (3.2) | 2.7 (6.8)  *P=*0.410 | 4.0 (8.4)  *P=*0.060 | 1.8 (6.0) |
| Change from baseline to week 8 | 4.0 (9.9)  *P=*0.677 | 2.6 (4.0)  *P=*0.850 | 2.2 (3.4) | 1.3 (4.5)  *P=*0.609 | 3.4 (8.1)  *P=*0.169 | 1.8 (6.1) |
| Change from baseline to week 12 | 3.2 (9.1)  *P=*0.767 | 3.0 (7.2)  *P=*0.761 | 1.8 (4.6) | 2.4 (6.1)  *P=*0.542 | 3.1 (7.3)  *P=*0.245 | 1.8 (5.7) |
| Change from baseline to week 16 | 2.0 (6.5)  *P=*0.905 | −1.0 (1.9)  *P=*0.088 | 2.4 (3.4) | 2.1 (7.3)  *P=*0.537 | 2.4 (6.7)  *P=*0.682 | 2.9 (7.2) |
| **Secondary, mean (SD) global SANDE scores** | | | | | | |
| Baseline | 82.6 (15.1) | 84.7 (11.4) | 77.9 (22.7) | 73.1 (16.1) | 72.5 (18.2) | 76.2 (13.9) |
| Change from baseline to week 4 (LOCF) | −17.7 (13.8)  *P=*0.516 | −22.8 (13.5)  *P=*0.207 | −12.8 (12.2) | −24.1 (27.2)  *P=*0.768 | −21.7 (24.9)  *P=*0.676 | −23.9 (22.0) |
| Change from baseline to week 8 | −23.0 (20.3)  *P=*0.611 | −27.9 (19.6)  *P=*0.377 | −17.4 (18.0) | −27.6 (26.4)  *P=*0.001^b^ | 17.4 (23.6)  *P=*0.531 | −16.8 (20.7) |
| Change from baseline to week 12 | −36.8 (13.2)  *P=*0.129 | −23.9 (18.1)  *P=*0.751 | −19.6 (23.1) | −26.9 (26.5)  *P=*0.005^b^ | −17.6 (24.0)  *P=*0.358 | −16.2 (19.5) |
| Change from baseline to week 16 | −42.8 (10.1)  *P=*0.011^b^ | −18.1 (18.1)  *P=*0.815 | −15.1 (20.5) | −24.8 (26.2)  *P=*0.046 | −18.8 (25.7)  *P=*0.792 | −16.8 (19.7) |
| **Secondary, mean (SD) severity of dryness and/or irritation SANDE scores** | | | | | | |
| Baseline | 78.0 (17.5) | 80.7 (13.2) | 70.2 (27.5) | 70.9 (17.5) | 70.7 (19.4) | 74.4 (15.4) |
| Change from baseline to week 4 | −16.7 (10.6)  *P=*0.086 | −21.4 (13.9)  *P=*0.054 | −7.2 (6.9) | −25.4 (28.2)  *P=*0.625 | −20.2 (25.5)  *P=*0.427 | −23.3 (22.7) |
| Change from baseline to week 8 | −20.9 (20.9)  *P=*0.313 | −22.8 (18.9)  *P=*0.228 | −10.5 (12.6) | −26.0 (28.4)  *P=*0.050 | −16.9 (25.3)  *P=*0.827 | −17.7 (21.8) |
| Change from baseline to week 12 | −33.1 (13.9)  *P=*0.036 | −19.2 (14.2)  *P=*0.439 | −10.8 (18.2) | −25.6 (27.7)  *P=*0.022^b^ | −15.0 (24.5)  *P=*0.709 | −16.3 (20.0) |
| Change from baseline to week 16 | −41.0 (11.5)  *P=*0.002^b^ | −14.0 (16.8)  *P=*0.561 | −7.8 (15.5) | −22.9 (27.6)  *P=*0.081 | −18.7 (26.6)  *P=*0.470 | −16.0 (19.7) |
| **Secondary, mean (SD) frequency of dryness and/or irritation SANDE scores** | | | | | | |
| Baseline | 87.6 (12.9) | 89.2 (10.8) | 87.8 (17.8) | 76.0 (16.6) | 74.8 (18.1) | 78.4 (14.0) |
| Change from baseline to week 4 | −18.7 (17.7)  *P=*0.919 | −23.6 (16.5)  *P=*0.752 | −19.8 (20.9) | −25.5 (27.0)  *P=*0.768 | −22.6 (26.0)  *P=*0.676 | −24.3 (24.1) |
| Change from baseline to week 8 | −25.1 (21.2)  *P=*0.939 | −33.2 (23.3)  *P=*0.652 | −26.2 (26.1) | −28.7 (26.7)  *P=*0.001^b^ | −17.5 (23.8)  *P=*0.531 | −15.1 (22.2) |
| Change from baseline to week 12 | −40.6 (13.7)  *P=*0.417 | −29.0 (22.7)  *P=*0.973 | −29.6 (30.8) | −27.9 (28.3)  *P=*0.005^b^ | −19.5 (24.9)  *P=*0.358 | −16.0 (21.4) |
| Change from baseline to week 16 | −43.7 (14.0)  *P=*0.154 | −22.4 (19.7)  *P=*0.891 | −24.6 (28.7) | −25.9 (27.7)  *P=*0.046 | −18.6 (26.0)  *P=*0.792 | −17.6 (21.8) |
| b.i.d., 2 times daily; LOCF, last observation carried forward; SANDE, Symptoms Assessment iN Dry Eye; SD, standard deviation; t.i.d., 3 times daily. *P* values calculated versus vehicle using *t* test. ^a^In the cenegermin b.i.d. group with patient with Sjögren’s dry eye disease, n=6 at baseline, from week 4 to end of study n=5; however, when LOCF is applicable (week 4 Schirmer I and global SANDE) n=6. ^b^Statistically significant (*P*<0.025) difference. | | | | | | |

| **Supplemental Table S4.** Summary of Change From Baseline on IDEEL Questionnaire by Presence of Sjögren’s | | | | | | |
| --- | --- | --- | --- | --- | --- | --- |
| **LS means (95% CI)** | **Patients with Sjögren’s dry eye disease** | | | **Patients with non-Sjögren’s dry eye disease** | | |
|  | **Cenegermin t.i.d.**  **N=7** | **Cenegermin b.i.d.**  **N=6** | **Vehicle**  **N=6** | **Cenegermin t.i.d.**  **N=74** | **Cenegermin b.i.d.**  **N=76** | **Vehicle**  **N=78** |
| **Daily activity limitations score** | | | | | | |
| Change from baseline to week 4 (LOCF) | 11.0  (−2.7, 24.6) | 18.9  (4.2, 33.6) | 14.4  (−0.3, 29.2) | 16.9  (12.3, 21.6) | 19.8  (15.2, 24.3) | 13.0  (8.5, 17.4) |
| Change from baseline to week 8 | 11.4  (−0.5, 23.3) | 19.9  (6.4, 33.3) | 6.7  (−6.2, 19.5) | 16.9  (12.1, 21.6) | 14.1  (9.6, 18.7) | 11.5  (7.0, 16.0) |
| Change from baseline to week 12 | 13.3  (−2.2, 28.8) | 17.2  (−0.4, 34.8) | 9.7  (−7.9, 27.3) | 15.1  (10.5, 19.8) | 15.2  (10.7, 19.7) | 10.8  (6.4, 15.2) |
| Change from baseline to week 16 | 12.4  (−2.5, 27.3) | 11.0  (−5.7, 27.8) | 9.4  (−7.4, 26.2) | 14.2  (9.5, 18.8) | 14.2  (9.7, 18.6) | 9.0  (4.7, 13.4) |
| **Emotional well-being score** | | | | | | |
| Change from baseline to week 4 | 21.1  (3.2, 39.0) | 12.9  (−6.5, 32.2) | 14.4  (−5.0, 33.8) | 13.6  (8.8, 18.5) | 16.6  (11.9, 21.3) | 15.1  (10.5, 19.7) |
| Change from baseline to week 8 | 21.8  (4.5, 39.0) | 15.7  (−3.4, 34.8) | 9.5  (−9.2, 28.1) | 15.1  (10.6, 19.6) | 12.7  (8.4, 17.0) | 13.0  (8.7, 17.2) |
| Change from baseline to week 12 | 23.1  (4.4, 41.7) | 11.8  (−8.8, 32.5) | 13.8  (−6.8, 34.4) | 12.8  (8.6, 17.0) | 15.0  (10.9, 19.0) | 14.0  (10.0, 18.0) |
| Change from baseline to week 16 | 25.6  (7.6, 43.7) | 11.0  (−9.0, 30.9) | 6.6  (−13.3, 26.4) | 14.7  (10.3, 19.2) | 15.1  (10.7, 19.4) | 13.7  (9.5, 18.0) |
| **Work limitations score** | | | | | | |
| Change from baseline to week 4 | 10.0  (−12.9, 32.9) | 40.0  (20.2, 59.8) | 12.2  (−8.5, 32.9) | 16.2  (8.3, 24.0) | 18.6  (10.7, 26.6) | 17.9  (10.8, 25.0) |
| Change from baseline to week 8 | 10.0  (−13.9, 33.9) | 26.3  (5.5, 47.0) | −0.9  (−22.0, 20.3) | 19.0  (11.8, 26.2) | 18.8  (11.3, 26.2) | 18.3  (11.8, 24.8) |
| Change from baseline to week 12 | 18.3  (−23.4, 60.0) | 27.5  (−7.8, 62.8) | 11.3  (−24.1, 46.6) | 16.8  (9.8, 23.9) | 20.8  (13.5, 28.1) | 18.3  (12.0, 24.6) |
| Change from baseline to week 16 | 11.8  (−18.9, 42.5) | 28.8  (2.7, 54.8) | 8.8  (−17.3, 34.8) | 19.0  (12.0, 26.0) | 20.2  (13.0, 27.5) | 16.2  (9.9, 22.5) |
| **Treatment satisfaction score** | | | | | | |
| Change from baseline to week 4 | 9.4  (−47.5, 66.3) | 21.9  (−11.0, 54.7) | 4.9  (−41.0, 50.9) | 24.4  (16.0, 32.8) | 19.7  (11.3, 28.2) | 26.3  (17.8, 34.8) |
| Change from baseline to week 8 | 9.4  (−43.2, 62.3) | 23.6  (−8.6, 55.7) | 7.8  (−29.6, 45.2) | 28.6  (20.8, 36.4) | 17.7  (10.3, 25.2) | 14.2  (6.5, 21.8) |
| Change from baseline to week 12 | 25.0  (−13.8, 63.8) | 24.5  (0.0, 49.0) | 1.4  (−28.1, 31.0) | 24.7  (16.8, 32.6) | 19.0  (11.5, 26.6) | 16.0  (8.3, 23.6) |
| Change from baseline to week 16 | 28.1  (−15.5, 71.8) | 25.9  (−1.3, 53.0) | −0.1  (−32.7, 32.6) | 25.4  (17.7, 33.1) | 22.9  (15.5, 30.2) | 14.2  (6.6, 21.7) |
| **Treatment-related bother score** | | | | | | |
| Change from baseline to week 4 | −3.3  (−42.1, 35.5) | 32.2  (0.6, 63.8) | 31.3  (−7.3, 69.8) | 15.4  (8.8, 22.0) | 16.1  (10.1, 22.1) | 13.2  (7.2, 19.3) |
| Change from baseline to week 8 | −7.8  (−48.0, 32.4) | 13.8  (−19.6, 47.1) | 23.4  (−16.8, 63.6) | 10.8  (4.3, 17.3) | 5.8  (−0.1, 11.7) | 4.1  (−1.8, 10.1) |
| Change from baseline to week 12 | −4.7  (−46.7, 37.3) | 16.0  (−18.6, 50.6) | 27.3  (−15.1, 69.7) | 9.7  (3.0, 16.4) | 7.1  (1.1, 13.1) | 8.0  (1.9, 14.1) |
| Change from baseline to week 16 | −9.4  (−53.0, 34.3) | 10.2  (−26.8, 47.2) | 33.8  (−10.2, 77.9) | 11.5  (5.3, 17.8) | 7.0  (1.4, 12.7) | 8.9  (3.2, 14.7) |
| **Symptom bother score** | | | | | | |
| Change from baseline to week 4 | −17.3  (−30.9, −3.7) | −12.3  (−27.0, 2.4) | −22.9  (−37.6, −8.2) | −16.5  (−20.5, −12.5) | −18.2  (−22.1, −14.3) | −16.0  (−19.9, −12.2) |
| Change from baseline to week 8 | −25.7  (−36.6, −14.8) | −12.8  (−25.5, −0.2) | −13.3  (−25.1, −1.5) | −20.1  (−24.2, −16.1) | −16.5  (−20.4, −12.6) | −13.5  (−17.3, −9.7) |
| Change from baseline to week 12 | −32.0  (−41.7, −22.2) | −14.2  (−25.7, −2.7) | −16.6  (−27.6, −5.6) | −17.7  (−21.5, −13.8) | −18.4  (−22.1, −14.7) | −12.5  (−16.2, −8.9) |
| Change from baseline to week 16 | −33.4  (−45.5, −21.3) | −11.9  (−26.1, 2.2) | −12.5  (−26.0, 1.0) | −18.2  (−22.0, −14.5) | −19.4  (−23.0, −15.7) | −10.9  (−14.4, −7.3) |
| b.i.d., 2 times daily; LOCF, last observation carried forward; IDEEL, Impact of Dry Eye on Everyday Life; LS, least squares; t.i.d., 3 times daily. Change from baseline in each IDEEL scale score (1 score for each questionnaire module) was analyzed using a mixed model for repeated measures, with fixed, categorical effects of treatment (cenegermin t.i.d., cenegermin b.i.d., and vehicle t.i.d.), visit (weeks 4, 8, 12, and 16), and treatment by visit interaction. Patient was considered as a random effect, and the covariance matrix used was “unstructured.” | | | | | | |

| **Supplemental Table S5.** Summary of TEAEs by Presence of Sjögren’s During the Treatment Period | | | | | | |
| --- | --- | --- | --- | --- | --- | --- |
| **n (%)** | **Patients with Sjögren’s dry eye disease** | | | **Patients with non-Sjögren’s dry eye disease** | | |
|  | **Cenegermin t.i.d.**  **N=9** | **Cenegermin b.i.d.**  **N=7** | **Vehicle**  **N=7** | **Cenegermin t.i.d.**  **N=76** | **Cenegermin b.i.d.**  **N=77** | **Vehicle**  **N=81** |
| Any TEAE | 8 (88.9) | 6 (85.7) | 2 (28.6) | 57 (75.0) | 54 (70.1) | 24 (29.6) |
| Any ocular TEAE | 8 (88.9) | 6 (85.7) | 1 (14.3) | 53 (69.7) | 54 (70.1) | 19 (23.5) |
| Any TEAE leading to discontinuation | 2 (22.2) | 1 (14.3) | 0 | 3 (3.9) | 1 (1.3) | 2 (2.5) |
| Any potentially related TEAE | 8 (88.9) | 6 (85.7) | 1 (14.3) | 52 (68.4) | 49 (63.6) | 12 (14.8) |
| Any serious TEAE | 0 | 0 | 0 | 0 | 0 | 0 |
| b.i.d., 2 times daily; TEAE, treatment-emergent adverse event; t.i.d., 3 times daily. | | | | | | |
