# Supplementary material for: Recombinant human nerve growth factor (cenegermin) for moderate-to-severe dry eye: phase II, randomized, vehicle-controlled, dose-ranging trial
Source: BMC Ophthalmol. 2024 Jul 17;24:290. doi: 10.1186/s12886-024-03564-w (PMC11253442; doi:10.1186/s12886-024-03564-w)
Supplement: Supplementary file 3 — Supplementary Material 3. [file 12886_2024_3564_MOESM3_ESM.pdf]

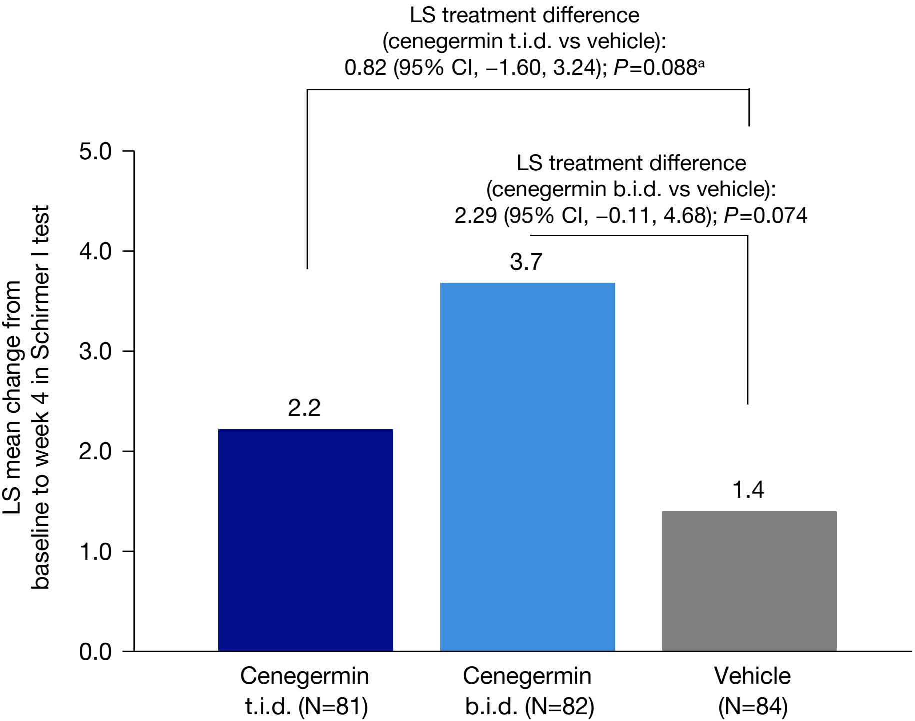

Supplemental Figure S1. Adjusted mean change from baseline in Schirmer I test at week 4 (LOCF, eligible eye; full analysis set). b.i.d., 2 times daily; LOCF, last observation carried forward; LS, least squares. [a]If the cenegermin t.i.d. dose was significantly different from vehicle, then the cenegermin b.i.d. dose was compared with vehicle; significance threshold was $P<0.025$.
