# Supplementary material for: Recombinant human nerve growth factor (cenegermin) for moderate-to-severe dry eye: phase II, randomized, vehicle-controlled, dose-ranging trial
Source: BMC Ophthalmol. 2024 Jul 17;24:290. doi: 10.1186/s12886-024-03564-w (PMC11253442; doi:10.1186/s12886-024-03564-w)
Supplement: Supplementary file 4 — Supplementary Material 4. [file 12886_2024_3564_MOESM4_ESM.pdf]

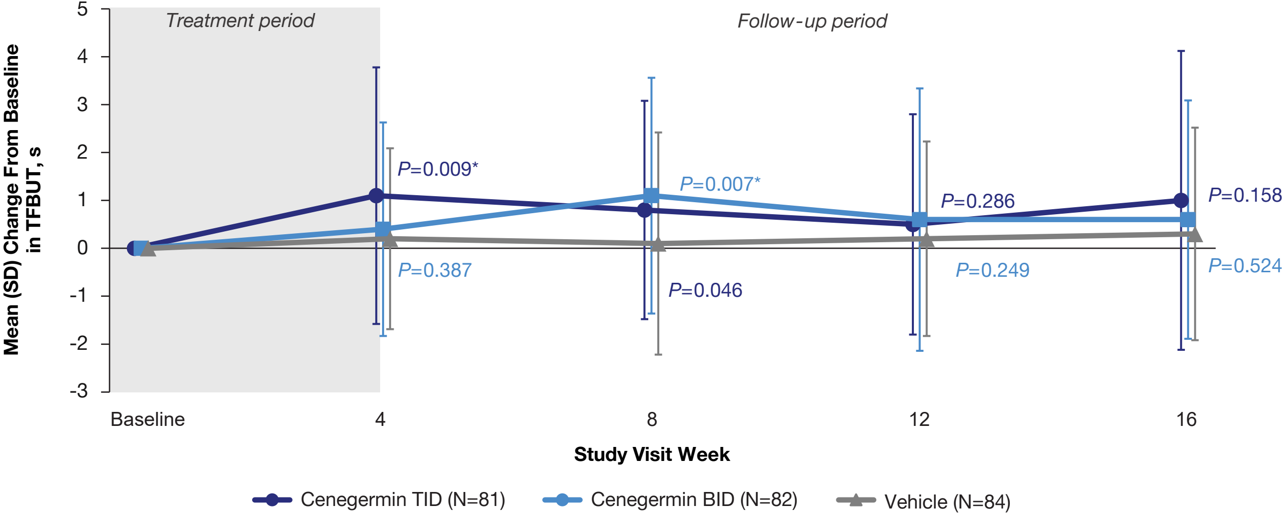

Supplemental Figure S2. Mean (SD) change from baseline in TFBUT by study visit (full analysis set). Change from baseline in TFBUT was analyzed at each time point using a *t* test for the comparison of cenegermin t.i.d. and b.i.d. vs vehicle. b.i.d., 2 times daily; SD, standard deviation; TFBUT, tear film break-up time; t.i.d., 3 times daily. *Denotes *P*<0.025.
