# Supplementary material for: Recombinant human nerve growth factor (cenegermin) for moderate-to-severe dry eye: phase II, randomized, vehicle-controlled, dose-ranging trial
Source: BMC Ophthalmol. 2024 Jul 17;24:290. doi: 10.1186/s12886-024-03564-w (PMC11253442; doi:10.1186/s12886-024-03564-w)
Supplement: Supplementary file 5 — Supplementary Material 5. [file 12886_2024_3564_MOESM5_ESM.pdf]

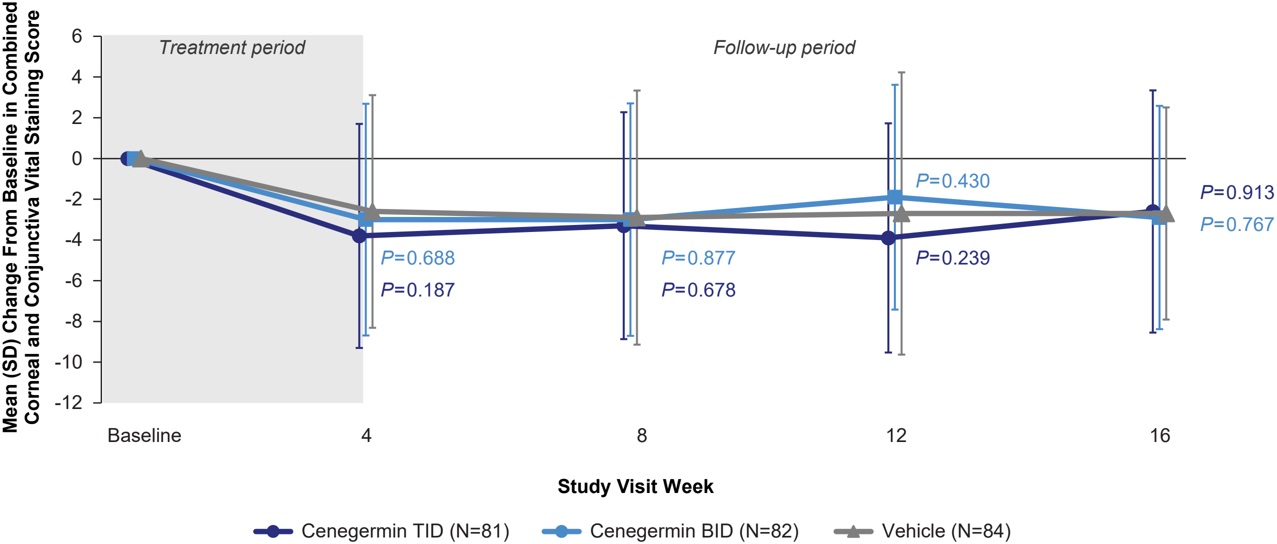

Supplemental Figure S3. Mean (SD) change from baseline in combined corneal and conjunctival vital staining score (assessed using the National Eye Institute scales) by study visit (full analysis set). Change from baseline to week 4 in corneal and conjunctival vital staining score was analyzed at each time point using a *t* test for the comparison of cenegermin t.i.d. and b.i.d. vs vehicle. b.i.d., 2 times daily; SD, standard deviation; t.i.d., 3 times daily.
